# High Thermoelectric Performance Achieved in Sb-Doped GeTe by Manipulating Carrier Concentration and Nanoscale Twin Grains

**DOI:** 10.3390/ma15020406

**Published:** 2022-01-06

**Authors:** Chao Li, Haili Song, Zongbei Dai, Zhenbo Zhao, Chengyan Liu, Hengquan Yang, Chengqiang Cui, Lei Miao

**Affiliations:** 1 School of Electromechanical Engineering, Guangdong University of Technology, Guangzhou 510006, China; 2 Ji Hua Laboratory, Foshan 528299, China; 3 The Fifth Electronics Research Institute of Ministry of Industry and Information Technology, Guangzhou 510006, China; dai_zongbei@126.com (Z.D.); zhenbozhao83@163.com (Z.Z.); 4 Key Laboratory of Bioinorganic and Synthetic Chemistry of Ministry of Education, School of Chemistry, Sun Yat-Sen University, Guangzhou 510275, China; 5 Guangxi Key Laboratory of Information Material, Guangxi Collaborative Innovation Center of Structure and Property for New Energy and Materials, School of Material Science and Engineering, Guilin University of Electronic Technology, Guilin 541004, China; chengyanliu@guet.edu.cn; 6 Jiangsu Key Laboratory of Modern Measurement Technology and Intelligent Systems, School of Physics and Electronic & Electrical Engineering, Huaiyin Normal University, Huai’an 223300, China; 7 SIT Research Laboratories, Innovative Global Program, Department of Materials Science and Engineering, Faculty of Engineering, Shibaura Institute of Technology, Tokyo 135-8548, Japan

**Keywords:** GeTe, Sb-doping, optimizing carrier concentration, nanoscale twin grains

## Abstract

Lead-free and eco-friendly GeTe shows promising mid-temperature thermoelectric applications. However, a low Seebeck coefficient due to its intrinsically high hole concentration induced by Ge vacancies, and a relatively high thermal conductivity result in inferior thermoelectric performance in pristine GeTe. Extrinsic dopants such as Sb, Bi, and Y could play a crucial role in regulating the hole concentration of GeTe because of their different valence states as cations and high solubility in GeTe. Here we investigate the thermoelectric performance of GeTe upon Sb doping, and demonstrate a high maximum zT value up to 1.88 in Ge_0.90_Sb_0.10_Te as a result of the significant suppression in thermal conductivity while maintaining a high power factor. The maintained high power factor is due to the markable enhancement in the Seebeck coefficient, which could be attributed to the significant suppression of hole concentration and the valence band convergence upon Sb doping, while the low thermal conductivity stems from the suppression of electronic thermal conductivity due to the increase in electrical resistivity and the lowering of lattice thermal conductivity through strengthening the phonon scattering by lattice distortion, dislocations, and twin boundaries. The excellent thermoelectric performance of Ge_0.90_Sb_0.10_Te shows good reproducibility and thermal stability. This work confirms that Ge_0.90_Sb_0.10_Te is a superior thermoelectric material for practical application.

## 1. Introduction

Thermoelectric (TE) materials can convert heat directly into available electricity based on the Seebeck effect, providing an alternative way to utilize fossil energy more efficiently [1,2,3,4,5,6]. The thermoelectric material performance can be estimated by the dimensionless figure of merit z*T* = *S*^2^*Tσ*/*κ*, where *S* is the Seebeck coefficient, *σ* is the electrical conductivity, *T* is the absolute temperature, and *κ* is the thermal conductivity, which usually stems from the crystal lattice vibrations (*κ_l_*) and the charge carriers drift (*κ_e_*). Hence, a good thermoelectric material should have a large *σ* and *S* along with a low *κ* [7].

In recent decades, IV–VI semiconductors with a narrow band gap have made a great progress in terms of theory and TE performance, showing a bright prospect in TE application [7,8,9,10,11]. These compounds exhibit a unique TE-favorable property portfolio such as high valley degeneracy, large dielectric function, and strong lattice anharmonicity empowered by metavalent bonding [12]. For example, the maximum *zT* exceeds 2.5 and it has been attained in PbTe and SnSe TE materials [13,14,15,16,17]. However, the toxicity of Pb and the easy cleavage of SnSe limit their commercialization [18]. Alternatively, GeTe is an eco-friendly counterpart in the family of IV–VI semiconductors, which also exhibits an excellent TE performance [18,19,20]. GeTe undergoes ferroelectric structural transition, transforming from low-temperature rhombohedral phase (R$\bar{3}$m, the primitive cell could be regarded as a slightly distorted cubic along <111> direction) into high-temperature cubic one (Fm$\bar{3}$m) with Curie temperature between 600 and 700 K [21]. It is believed that such a slight symmetry breaking is conducive to converging the splitting valence bands and reducing the *κ_l_* [22,23]. Additionally, some elements such as Sb, Bi, and Y generally have a high doping limit in GeTe, for instance, the solubility of Sb exceeds 10% in GeTe. Undoubtedly, such features are beneficial to adequately optimize the electronic transport properties, and, in turn, yield a high *zT*, e.g., the maximum *zT* over 2 is attainable in (Bi, Cu), (Bi, Pb), (Sb, Pb), and (Sb, In) codoping systems [24,25,26,27,28].

However, the pristine GeTe commonly exhibits an inferior TE performance due to its low *S* and high *κ*, i.e., the large number of Ge vacancies causes a high hole concentration (~10^21^ cm^−3^), a low *S* (~34 μV K^−1^), and a high *κ* (~8 W m^−1^ K^−1^) at room temperature [29]. To suppress Ge vacancies and hole concentration, Cu interstitials and Sb/Bi/Y substitutions were introduced into GeTe matrix to compensate for the excess holes or increase the formation energy of Ge vacancy. For example, the hole concentration approaching ~10^20^ cm^−3^ could be obtained by Sb/Bi/Y doping, and the available maximum *zT* is up to 1.7–1.85 [30,31,32,33]. Aside from holes concentration optimization, the resonant doping and valence band alignment could also increase the *S*. It has been demonstrated that In/Ga/Ti doping could introduce the resonant level around the Fermi level [34,35,36,37], and Mn/Cd/Zn doping could decrease the split energy between light and heavy valence bands for GeTe [22,23,25]. In practice, the strategy that combines carrier concentration optimization with band alignment or resonant doping has been extensively used to optimize the electronic transport properties [34,35,38,39].

In addition to the optimization of electrical transport properties, doping or alloying can introduce significant lattice disorder, resulting in atomic mass and lattice strain fluctuations that endow a low *κ_l_* by the reinforcement of phonon scattering [40]. For example, the room temperature *κ_l_* of pristine GeTe (2.7 W m^−1^ K^−1^) can be decreased within the range of 1.0–1.5 W m^−1^ K^−1^ by doping 10 at% Sb/Bi/Pb [41,42]. This value can even be further suppressed to ~0.5 W m^−1^ K^−1^ in codoping systems, for instance, Ge_0.88__−*y*_Sb_0.08_Pb*_y_*Te and Ge_0.85_Bi_0.05_Sb_0.10_Te alloys [43]. This prominent reduction in *κ_l_* was mainly attributed to the atomic mass and lattice strain fluctuations induced by point defects. Yet, with the substitution of extrinsic atoms on Ge sites, the evolution of microstructures is complicated, often involving precipitates [27,31,44,45], stacking faults [42,46,47], inversion domain and twin [21,31,48,49,50], as well as herringbone domain structures [48,51]. These structural defects should also play an important role in strengthening the phonon scattering. Overall, doping/alloying is an imperative strategy for the enhancement of TE performance of GeTe through regulating the electron and phonon transport properties via energy band and defects engineering.

Here, we systematically investigated the TE performance of Ge_1−*x*_Sb*_x_*Te (*x* = 0, 0.05, 0.10, 0.15, and 0.20), which were prepared by the solid-state reaction and spark plasma sintering (SPS) route. We proved that a maximum *zT* of 1.88 at 773 K could be attained in Ge_0.90_Sb_0.10_Te with good reproducibility and thermal stability. With Sb substitution on Ge sites, the *Seebeck* is greatly improved due to the reduction in hole concentration and valence band convergence. Moreover, a significantly reduced *κ* is achieved, which originates from both the decrease in *κ_e_* and *κ_l_*. Transmission electron microscopy (TEM) observations verify the existence of nanoscale twins and dislocations in the matrix, which together with point defects (Sb substitutions and residual Ge vacancies) result in a low *κ_l_*, i.e., ~0.89 W m^−1^ K^−1^ in Ge_0.90_Sb_0.10_Te at 773K. This work confirms Ge_0.90_Sb_0.10_Te is a reliable candidate for TE application.

## 2. Materials and Methods

Polycrystalline samples Ge_1−*x*_Sb*_x_*Te (*x* = 0, 0.05, 0.10, 0.15, 0.20) were prepared through a solid-state reaction followed by spark plasma sintering (SPS) procedure. High-purity (>4 N) elements of Ge, Sb, and Te were weighted in the corresponding mole ratio and flame-sealed in quartz tubes under a vacuum lower than 5 × 10^−4^ Pa. The tubes were heated to 1173 K and soaked for 6 h, both of heating and cooling rate at 1 K min^−1^. These as-synthesized ingots were hand-ground into fine powders and then consolidated into cylindrical bulks by SPS at 823 K for 3 min under a uniaxial pressure of ~50 MPa in a vacuum (˂10 Pa). The relative density for these sintered samples is no less than 95%. Rectangular specimens (~2 mm × 2 mm × 10 mm) and square-shaped pellets (~6 mm × 6 mm × 1.5 mm) were cut off for electrical and thermal transport properties measurements, respectively. 

The crystal structure of sintered samples was analyzed using a PANalytical X-ray diffractometer (Cu K*α*, λ = 0.154 nm) operated at 45 kV and 40 mA with a step size of 0.01313°. The high-angle annular dark field scanning transmission electron microscopy (HAADF-STEM) and the energy-dispersive X-ray (EDS) mapping were performed on a probe-corrected transmission electron microscopy (TEM; FEI Titan G2) equipped with a super EDS system at 300 kV. The backscattered electron image (BSE) and EDS mapping scanning were employed with electron microscopy (SEM, FEI Helos G4) equipped with a super EDS system.

Room temperature carrier concentration (*p*) and mobility (μ_H_) were examined using a direct-current (*dc*) Hall effect measurement system (model 8404; Lakeshore Cryotronics, USA) at the excitation current up to 100 mA and magnetic field of 1.5 T. Electrical resistivity (*ρ*) and Seebeck coefficient (*S*) were measured by a static *dc* method (LSR-3, Germany) under a helium (99.999%) atmosphere. The thermal conductivity (*κ*) is calculated by *κ* = *D d C_p_*, where *D* is the thermal diffusivity, *C_p_* is the specific heat, and *d* is the volume density. The thermal diffusivity (*D*) was measured by a laser flash method (LFA 457; Netzsch, Germany); the specific heat (*C_p_*) was estimated based on Dulong–Petit law, which is approximate to the measurement result; and the volume density (*d*) was determined by the Archimedes method.

## 3. Results and Discussion

**Figure 1:**
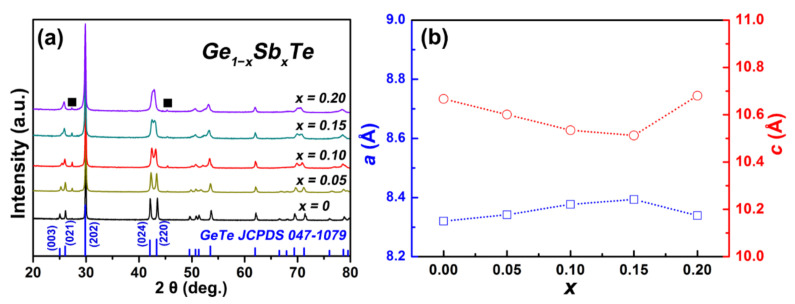
(**a**,**b**) Powder XRD patterns and lattice parameters *a* and *c* of Ge_1−*x*_Sb*_x_*Te. The black square (■) represents Ge precipitates.

Figure 1 shows the XRD patterns of the as-pulverized sintered Ge_1−*x*_Sb*_x_*Te samples. The major diffraction peaks of each sample could be indexed to the rhombohedral structure of GeTe (JCPDS 047−1079). Particularly, the presence of double peaks in the 2θ range of 23–27° and 41–45° further confirms the rhombohedral phase of pristine GeTe. The peaks located at ~27.3° and ~45.3° correspond to cubic Ge (JCPDS 065−0333) secondary phase. The obviously enlarged Ge peaks with increasing the Sb content indicates more Ge precipitates in the matrix, which is probably due to that Sb atoms occupy on Ge sublattice sites. To examine the impact of Sb doping on the crystal structure of GeTe, we calculated lattice parameters of the sintered Ge_1−*x*_Sb*_x_*Te samples and the results are shown in Figure 1b. We observed the increase in lattice parameter *a* and decrease in *c* with increasing the Sb content, suggesting a smaller *c*/*a* ratio. This phenomenon indicates that Sb doping could lower the structural transition temperature of GeTe and promote the formation of cubic phase, which is consistent with the convergence of (024) and (220) diffraction peaks (Figure 1a), and the observations in the Bi- and V-doped cases [32,47].

**Figure 2:**
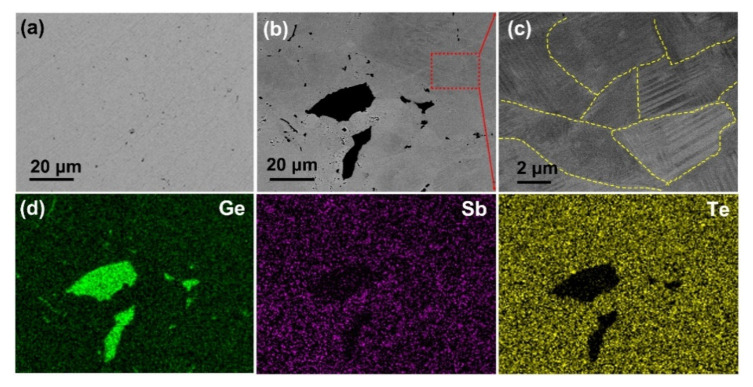
BSE images of the pristine GeTe (**a**) and Ge_0.90_Sb_0.10_Te (**b**); (**c**) magnified ECC image of Ge_0.90_Sb_0.10_Te taken on the region marked by the red dotted rectangle in Figure 2b; (**d**) elemental mapping images taken on the region that displayed in Figure 2b.

We performed SEM and EDS to illustrate the microstructure evolution of GeTe upon Sb doping, as shown in Figure 2. Figure 2a shows a BSE image of the pristine GeTe, it can be seen that a small amount of nanoscale precipitates (dark contrast) is embedded into the host matrix (gray contrast). A BSE image of Ge_0.90_Sb_0.10_Te (Figure 2b) shows that there are many micron-sized precipitates (dark contrast) in the matrix. The EDS mapping images for Ge_0.90_Sb_0.10_Te shown in Figure 2d unveil that the precipitates are Ge particles, which agree with the XRD characterizations. Moreover, the electron channeling contrast image (ECCI) is taken on the region marked with the red dotted rectangle to characterize the microstructure of the matrix [52], as shown in Figure 2c, which suggests that the matrix is comprised by 2–10 micron-sized grains, and each grain includes several lamellar configurations of the size of 20–500 nm.

**Figure 3:**
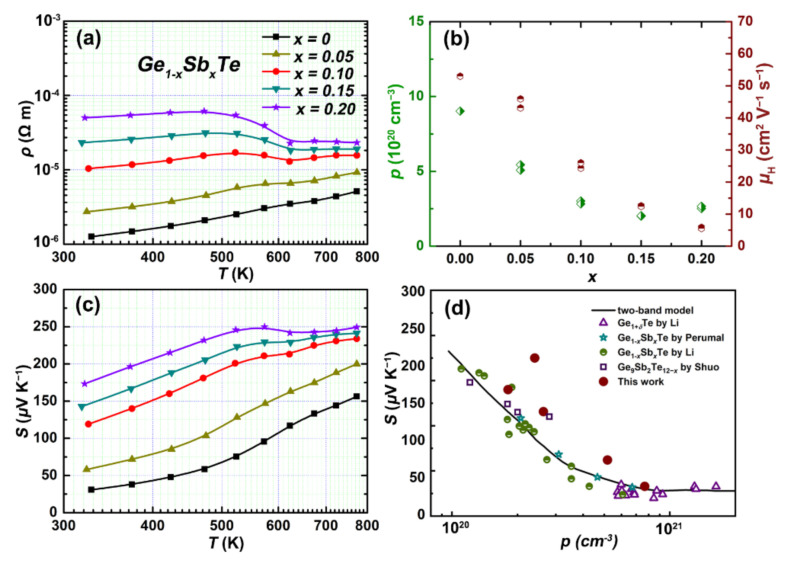
Electrical properties of Ge_1__−*x*_Sb*_x_*Te: (**a**) electrical conductivity; (**b**) carrier concentration and mobility as a function of the Sb content *x* at room temperature; (**c**) Seebeck coefficient; (**d**) Pisarenko plot.

The electrical transport properties of the samples Ge_1−*x*_Sb*_x_*Te are shown in Figure 3. From Figure 3a, we can see that the resistivity *ρ* of all the samples increases with rising temperature, showing a degenerate semiconductor behavior, coincides with its incipient metal nature of GeTe empowered by metavalent bonding [53]. Typically, for the pristine GeTe, the *ρ* is ~1.26 × 10^−6^ Ω m at 323 K, which increases to ~5.11 × 10^−6^ Ω m at 773 K. As Sb substitution on Ge sites could generate a donor type point defect and increase the formation energy of Ge vacancy, the *ρ* increases with increasing the Sb content. For example, the *ρ* at 323 K increases from ~1.26 × 10^−6^ Ω m for the pristine GeTe to 5.01 × 10^−5^ Ω m for the sample Ge_0.80_Sb_0.20_Te. The abnormal change in *ρ*~*T* curves shows a signature of structural phase transition. The transition temperature could be shifted to a lower temperature with increasing the Sb content, which is also observed in the Bi-doped GeTe system. This makes it possible to enhance the TE performance by controlling the distortion degree of the crystal structure from cubic to rhombohedral [39].

The Hall measurement was carried out on the samples Ge_1__−_*_x_*Sb*_x_*Te, as shown in Figure 3b. It clearly shows that the carrier concentration and mobility are significantly suppressed upon Sb doping, mainly due to the generation of the charged substitutional point defects. For example, the room temperature carrier concentration gradually declines from ~9.02 × 10^20^ to ~2.01 × 10^20^ cm^−3^ as the Sb content increases from *x* = 0 to *x* = 0.15, then rises to ~2.55 × 10^20^ cm^−3^ when *x* = 0.20, indicating that the doping limit of Sb in GeTe is about 15 at%, and the doping efficiency is greatly reduced when the Sb content exceeds 10 at% [43,50]. The room-temperature carrier mobility monotonically drops from 53 cm^2^ V^−1^ s^−1^ for the pristine GeTe to 5.9 cm^2^ V^−1^ s^−1^ for the sample Ge_0.80_Sb_0.20_Te. Both the declined carrier concentration and mobility lead to the increase in *ρ* for the Sb-doped GeTe system.

Figure 3c shows the temperature-dependent Seebeck coefficient (*S*) of the samples Ge_1−*x*_Sb*_x_*Te. The positive *S* suggests that all the samples have a p-type conductive behavior and the inability of single Sb doping in inversing such a conductive behavior. As expected, the *S* is greatly improved upon Sb doping, increasing from ~31 μV K^−1^ for the pristine GeTe to ~119 μV K^−1^ for Ge_0.90_Sb_0.10_Te, and further to ~173 μV K^−1^ for Ge_0.80_Sb_0.20_Te at 323 K. A maximum *S* up to 249 μV K^−1^ at 574 K is attained in Ge_0.80_Sb_0.20_Te.

To find out the underlying reason for the enhanced *S* of GeTe upon Sb doping, the Pisarenko curve based on a two-valence-band model is plotted in Figure 3d, and the representative data from the literature are given for comparison. It can be seen that the data for the pristine GeTe fall exactly on the Pisarenko curve, while the data for the Sb-doped samples lie above the curve, indicating that the valence band convergence could be induced by Sb doping. According to the results of band structure calculations, the maximum of the heavy valence band is higher than that of the light one for the rhombohedral GeTe, which inverses for the cubic GeTe [31]. As mentioned above, Sb doping promotes the structural transition of GeTe from rhombohedral to cubic, leading to band convergence by decreasing the split energy between the heavy and light valence bands. Overall, the greatly enhanced *S* in the Sb-doped GeTe samples could be attributed to the valence band convergence and the hole concentration suppression, as the *S* is inversely coupled with the carrier concentration.

Figure S1 shows the temperature-dependent power factor (*PF*) of the samples Ge_1−*x*_Sb*_x_*Te. The maximum *PF* as high as ~4.74 × 10^−^^3^ W m^−1^ K^−2^ at 773 K is attained in the pristine GeTe. The high *PF* is maintained when the Sb content does not exceed 10 at% due to the greatly enhanced *S*, despite the evident increase in *ρ*. The maximum *PF* up to ~3.23 × 10^−^^3^ W m^−1^ K^−2^ achieved in Ge_0.90_Sb_0.10_Te is comparable to V-, Y-, Bi-, and Sb-doped counterparts [30,32,33,47].

**Figure 4:**
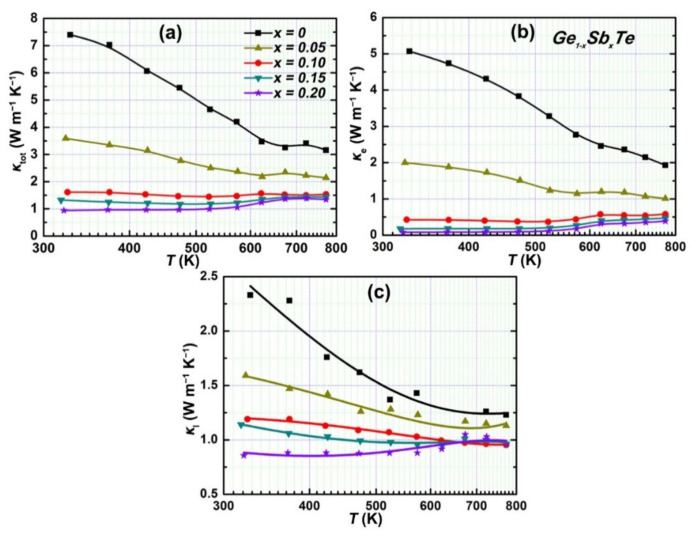
Temperature-dependent thermal properties of Ge_1__−*x*_Sb*_x_*Te: (**a**) thermal conductivity; (**b**) electronic thermal conductivity; (**c**) lattice thermal conductivity.

Figure 4a shows the temperature-dependent thermal conductivity (*κ*) of the samples Ge_1__−*x*_Sb*_x_*Te. The *κ* is greatly reduced over the whole temperature range (323–773 K) upon Sb doping, which decreases from ~7.40 W m^−1^ K^−1^ for the pristine GeTe to 1.61 W m^−1^ K^−1^ for the sample Ge_0.90_Sb_0.10_Te at 323 K. To understand the details regarding the great reduction of *κ*, the *κ* is generally divided into two parts of electronic thermal conductivity (*κ_e_*) and lattice thermal conductivity (*κ_l_*). The *κ_e_* is calculated according to the Wiedemann–Franz law, *κ_e_* = *L ρ*^−1^ T, where *L* is the Lorentz number estimated based on a two-valence-band model (Figure S2). As shown in Figure 4b, it is observed that the *κ_e_* for GeTe could be remarkably suppressed upon Sb doping due to the significant increase in *ρ*. For example, the *κ_e_* as high as 5.07 W m^−1^ K^−1^ at 323 K is attained in the pristine GeTe, which dramatically drops to 0.43 W m^−1^ K^−1^ for Ge_0.90_Sb_0.10_Te. The *κ_l_* is obtained by subtracting *κ_e_* from *κ*, i.e., *κ_l_* = *κ* – *κ_e_*. The temperature-dependent *κ_l_* of the Ge_1−*x*_Sb*_x_*Te is plotted in Figure 4c. It indicates that the *κ_l_* of GeTe could be substantially suppressed upon Sb doping, e.g., the highest *κ_l_* up to ~2.33 W m^−1^ K^−1^ at 323 K is attained in the pristine GeTe, which decreases to ~1.19 W m^−1^ K^−1^ for the sample Ge_0.90_Sb_0.10_Te.

**Figure 5:**
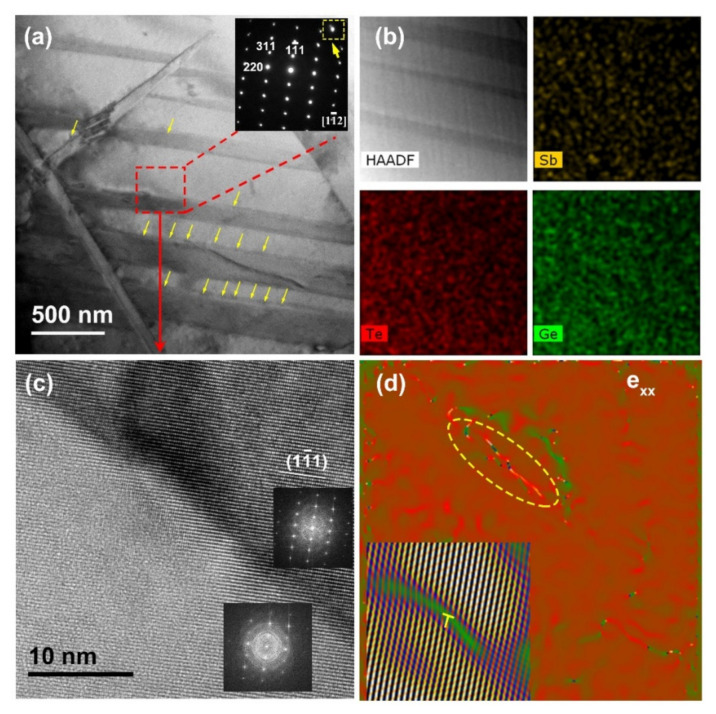
The microstructure characterization of Ge_0.90_Sb_0.10_Te: (**a**) a low-magnification bright-field image, the inset is the SAED pattern taken on the marked region; (**b**) a HADDF image and the corresponding elemental mappings; (**c**) a HRTEM image from the marked region in Figure 5a, the insets are the corresponding FFT images; (**d**) the distribution of strain calculated by GPA, the inset is the corresponding IFFT image.

To illustrate the low *κ_l_* of Ge_0.90_Sb_0.10_Te, its microstructural details were investigated by TEM. A typical bright-field TEM image, as shown in Figure 5a, reveals the stacking lamellae in alternate bright and dark contrast, and there exists fluctuation in contrast at the edges of the lamellae (marked with the yellow arrows). However, the elemental Ge, Sb, and Te are uniformly distributed in the host matrix according to the EDS mapping results (Figure 5b), suggesting that the change in bright and dark contrast is unrelated to chemical compositions. A typical selected area electron diffraction (SAED) pattern of the lamellar structures can only be indexed to the [1$\bar{1}$2] zone axis with rhombohedral symmetry (see the inset in Figure 5a). A HRTEM image is obtained in the same zone axis, which matches with the lattice fringes of the (111) planes of rhombohedral GeTe (Figure 5c). Moreover, the split diffraction spots far away from the transmitted beam are observed in the SAED pattern, together with the fast Fourier transform (FFT) image (the inset in Figure 5c), demonstrating that the lamellar structure is caused by the twin grains, which is similar to the herringbone structure observed in Bi-/Sb-doped GeTe samples [30,32]. It has been demonstrated that twin boundaries can effectively scatter phonons while maintaining a high charge carrier mobility [54]. Furthermore, the inverse FFT (IFFT) image discloses that there are dislocations at the twin boundaries, resulting from the release of strain. Additionally, geometric phase analysis (GPA) is executed to calculate the distribution of strain in the matrix based on the HRTEM image (Figure 5c), as shown in Figure 5d, confirming that an obvious strain fluctuation distributes around the dislocations. It is known that dislocations are very conducive to reducing the *κ_l_* by scattering the mid-frequency phonons [52]. Overall, the low *κ_l_* of Ge_0.90_Sb_0.10_Te could be attributed to its complex microstructures such as point defects (Sb substitutions and residual Ge vacancies), dislocations, and twin boundaries.

**Figure 6:**
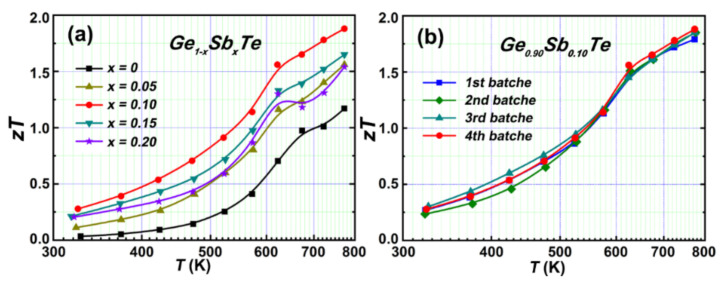
(**a**) *zT* value of Ge_1__−*x*_Sb*_x_*Te (*x* = 0, 0.05, 0.10, 0.15, 0.20); (**b**) *zT* value of the different batch samples Ge_0.90_Sb_0.10_Te.

The TE performance of GeTe is greatly enhanced upon Sb doping as a result of holes concentration optimization, band convergence, and the significant decrease in thermal conductivity. A highest *zT* up to 1.88 is achieved in the sample Ge_0.90_Sb_0.10_Te (Figure 6a), which is higher than Bi-/In-/Ti-doped counterparts (single extrinsic elemental doping systems) [32,37,55]. It demonstrates that elemental Sb is an outstanding dopant for enhancing the TE performance of GeTe.

More importantly, the high TE performance of Ge_0.90_Sb_0.10_Te is reproducible, which is verified by the repeatable *zT* of the different baches samples, as shown in Figure 6b. Additionally, the thermal cycling test was performed on a sample Ge_0.90_Sb_0.10_Te, the electrical properties remain unchanged during the 2 times heating and cooling cycles (Figure S3), suggesting excellent stability of Ge_0.90_Sb_0.10_Te. These results indicate Ge_0.90_Sb_0.10_Te has promising application in commercial TE devices.

## 4. Conclusions

The Ge_1−*x*_Sb*_x_*Te samples were fabricated by the solid-state reaction followed by spark plasma sintering procedure. Sb doping not only leads to an optimized hole concentration approaching 10^20^ cm^−3^ but also results in valence band convergence for the GeTe system. Moreover, the thermal conductivity is greatly reduced due to the suppression of both electronic and lattice thermal conductivity. While the reduction in electronic thermal conductivity is due to the increase of electrical resistivity, the decline in lattice thermal conductivity is caused by the modification in microstructures, that is, the phonon scattering is remarkably strengthened by substitutional point defects, dislocations, and twin boundaries. As a result, a maximum *zT* up to 1.88 is achieved in Ge_0.9_Sb_0.1_Te. This work demonstrates that Ge_0.90_Sb_0.10_Te is a promising candidate for TE commercial application.

## Data Availability

The data presented in this study are available on request from the corresponding author.

## Supplementary Materials

The following are available online at https://www.mdpi.com/article/10.3390/ma15020406/s1, this article at Figure S1: Temperature-dependent power factor of Ge_1−x_Sb_x_Te (x = 0, 0.05, 0.10, 0.15, 0.20), Figure S2: Temperature-dependent Lorentz number of Ge_1−x_Sb_x_Te (x = 0, 0.05, 0.10, 0.15, 0.20), and Figure S3: Temperature-dependent electrical properties of the Ge_0.90_Sb_0.10_Te under thermal cycles: (a) electrical conductivity; (c) Seebeck coefficient.
