# Supplementary material for: High Thermoelectric Performance Achieved in Sb-Doped GeTe by Manipulating Carrier Concentration and Nanoscale Twin Grains
Source: Materials (Basel). 2022 Jan 6;15(2):406. doi: 10.3390/ma15020406 (PMC8777978; doi:10.3390/ma15020406)
Supplement: Supplementary file 1 [file materials-15-00406-s001.zip › materials-1453858-supplementary.pdf]

Supplementary

High Thermoelectric Performance Achieved in Sb-doped GeTe by Manipulating Carrier Concentration and Nanoscale Twin Grains

Chao Li[1,2,3,¶], Haili Song[4,¶], Zongbei Dai[3,¶], Zhenbo Zhao[3], Chengyan Liu[5], Hengquan Yang[6,*], Chengqiang Cui[1,2,*], Lei Miao[5,7,*]

[1]School of Electromechanical Engineering, Guangdong University of Technology, Guangzhou, Guangdong 510006, China

[2]Ji Hua Laboratory, Foshan, Guangdong 528299, China

[3]The Fifth Electronics Research Institute of Ministry of Industry and Information Technology, Guangzhou, Guangdong 510006, China

[4]Key Laboratory of Bioinorganic and Synthetic Chemistry of Ministry of Education, School of Chemistry, Sun Yat-Sen University, Guangzhou, Guangdong 510275, China.

[5]Guangxi Key Laboratory of Information Material, Guangxi Collaborative Innovation Center of Structure and Property for New Energy and Materials, School of Material Science and Engineering, Guilin University of Electronic Technology, Guilin, 541004, P. R. China.

[6]School of Physics and Electronic & Electrical Engineering, and Jiangsu Key Laboratory of Modern Measurement Technology and Intelligent Systems, Huaiyin Normal University, Huai'an, 223300, P. R. China

[7]Department of Materials Science and Engineering, SIT Research Laboratories, Innovative Global Program, Faculty of Engineering,

Shibaura Institute of Technology, Tokyo 135-8548, Japan

* Corresponding author: Chengqiang Cui cqcui@gdut.edu.cn; Lei Miao miaolei@guet.edu.cn; Quanheng Yang yhq@hytc.edu.cn

¶ These authors contributed equally to this work

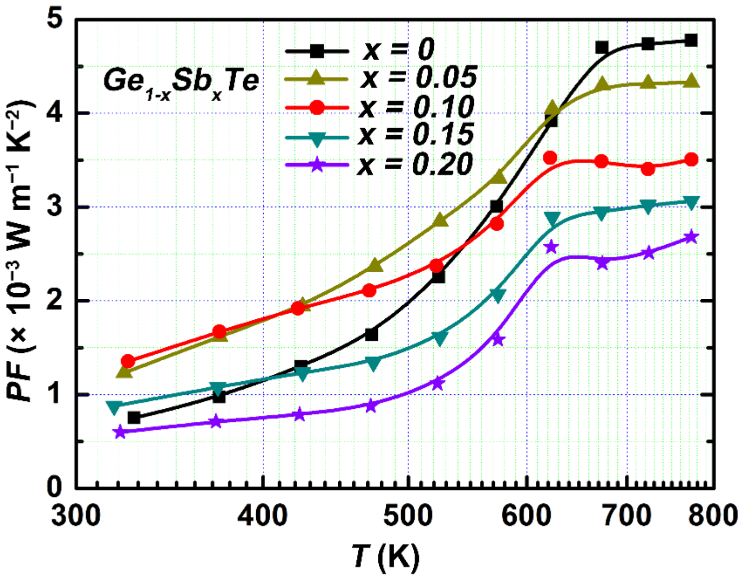

Figure S1 Temperature-dependent power factor of $Ge_{1-x}Sb_xTe$ (x = 0, 0.05, 0.10, 0.15, 0.20).

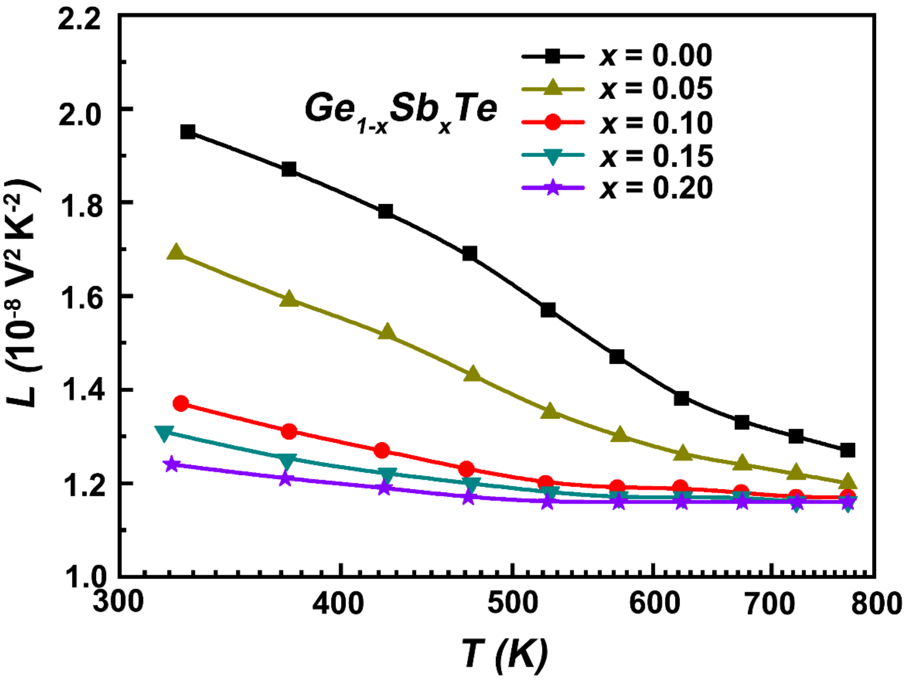

Figure S2 Temperature-dependent Lorentz number of $Ge_{1-x}Sb_xTe$ (x = 0, 0.05, 0.10, 0.15,

0.20), which is calculated based on a two-valence-band model [1].

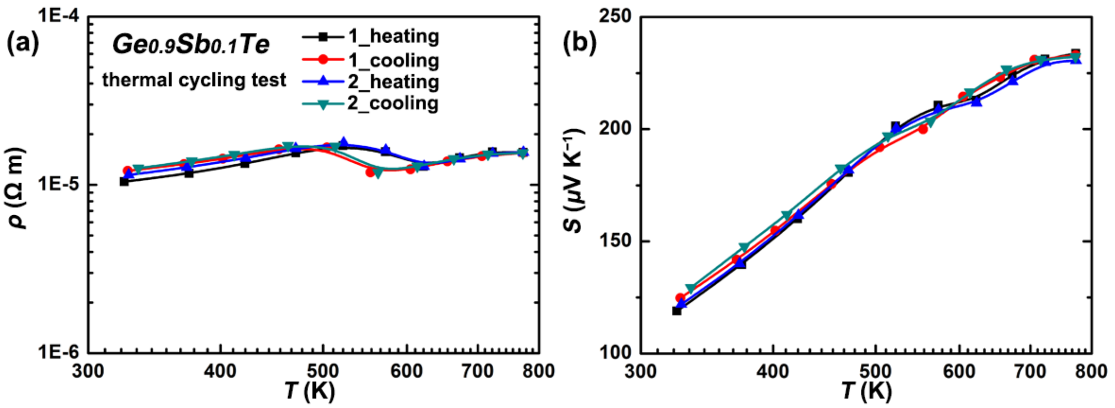

Figure S3 Temperature-dependent electrical properties of the $Ge_{0.90}Sb_{0.10}Te$ under thermal cycles: (a) electrical conductivity; (c) Seebeck coefficient.

Reference

[1] Zhang X, Li J, Wang X, et al. Vacancy Manipulation for Thermoelectric Enhancements in GeTe Alloys. J Am Chem Soc 2018, **140**: 15883−15888.
